# Robust and orange-yellow-emitting Sr-rich polytypoid α-SiAlON (Sr_3_Si_24_Al_6_N_40_:Eu^2+^) phosphor for white LEDs

**DOI:** 10.1080/14686996.2024.2396276

**Published:** 2024-09-02

**Authors:** Mehdi Estili, Rong-Jun Xie, Kohsei Takahashi, Shiro Funahashi, Tohru S. Suzuki, Naoto Hirosaki

**Affiliations:** a Research Center for Electronic and Optical Materials, National Institute for Materials Science (NIMS), Tsukuba, Ibaraki, Japan; b College of Materials, Xiamen University, Xiamen, China

**Keywords:** White light emitting diodes, phosphors, nitrides, photoluminescence, powder synthesis

## Abstract

Nitrides and oxynitrides isostructural to α-Si_3_N_4_ (*M*-α-SiAlON, *M* = Sr, Ca, Li) possess superb thermally stable photoluminescence (PL) properties, making them reliable phosphors for high-power solid-state lighting. However, the synthesis of phase-pure Sr-α-SiAlON still remains a great challenge and has only been reported for Sr below 1.35 at.% as the large size of Sr^2+^ ions tends to destabilize the α-SiAlON structure. Here, we succeeded to synthesize the single-phase powders of a unique ‘Sr-rich’ polytypoid α-SiAlON (Sr_3_Si_24_Al_6_N_40_:Eu^2+^) phosphor with three distinctive Sr/Eu luminescence sites using a solid-state remixing-reannealing process. The Sr content of this polytypoid structure exceeds those of a few previously reported structures by over 200%. The phase purity, composition, structure, and PL properties of this phosphor were investigated. A single phase can be obtained by firing the stoichiometric mixtures of all-nitride precursors at 2050°C under a 0.92 MPa N_2_ atmosphere. The Sr_3_Si_24_Al_6_N_40_:Eu^2+^ shows an intense orange-yellow emission, with the emission maximum of 590 nm and internal/external quantum efficiency of 66%/52% under 400 nm excitation. It also has a quite small thermal quenching, maintaining 93% emission intensity at 150°C. In comparison to Ca-α-SiAlON:Eu^2+^, this Sr counterpart shows superior quantum efficiency and thermal stability, enabling it to be an interesting orange-yellow down-conversion luminescent material for white LEDs. The experimental confirmation of the existence of such ‘Sr-rich’ SiAlON systems, in a single-phase powder form, paves the way for the design and synthesis of novel ‘Sr-rich’ SiAlON-based phosphor powders with unparalleled properties.

## IMPACT STATEMENT

A distinctive orange-yellow-emitting ‘Sr-rich’ α-SiAlON-based phosphor with quite small thermal quenching (93% PL intensity at 150°C) that can surprisingly be synthesized in a single-phase powder form for white LEDs.

## GRAPHICAL ABSTRACT

**Figure:**
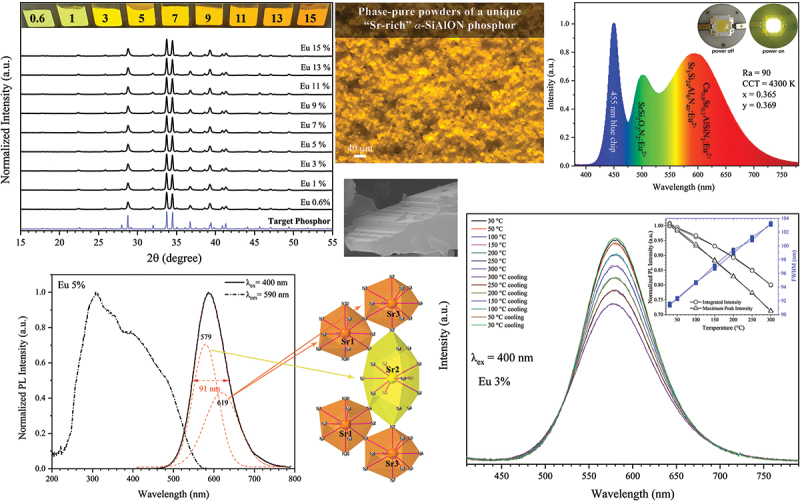

## 1. Introduction

Since their initial market introduction in autumn 1996 [1,2], white light-emitting diodes (wLEDs) have established themselves as reliable compact solid-state luminescent devices that promise ever-increasing energy conversion efficiency, long lifetime, high output power, and acceptable environmental compatibility. Therefore, they have found valuable applications in general lighting and display technologies [3–9]. In principle, white light can be created purely via electroluminescence (EL) using complex combinations of multiple GaN- or InGaN-based LED chips [10,11]. However, designing GaN- or InGaN-based semiconductor materials and emitting devices with desired emission colors, high efficiency, and stable performance simultaneously is undeniably challenging because it demands both effective charge transport properties and efficient emissions [12]. Furthermore, they usually provide poor color rendering as the generated white light is comprised of rather narrow emissions [13]. A feasible solution is to pump phosphors with ultraviolet- or blue-LED chips, forming the so-called phosphor-converted wLEDs (pc-wLEDs). This technology allows for the fabrication of cost-effective wLEDs with controllable color rendition [12–20].

Numerous phosphors with diverse optical properties and emission colors were designed or discovered using different host crystals and activator ions. They can be divided into prime classes of garnets [21,22], orthosilicates [23–25], phosphates [26–29], molybdates [30,31], vanadates [27,32–35], fluorides [35,36], oxyfluorides [37,38], sulfides [39,40], oxysulfides [41,42], and nitrides/oxynitrides [15,16,43,44]. Covalent nitrides and oxynitrides, which possess 2D or 3D networks of condensed edge- and/or corner-sharing, or face-sharing, or isolated Si(N/O)_4_, Al(N/O)_4_, Mg(N/O)_4_, Li(N/O)_4_, Ga(N/O)_4_, or Ge(N/O)_4_ tetrahedral building blocks, are widely recognized as the ultimate host materials for designing practical phosphors for solid-state lighting (SSL) applications. Their stiff, stable, and diverse structures and chemistries [45] generally result in thermally stable PL characteristics in addition to red-shifted emission and excitation spectra, abundant emission colors (from blue to deep red), acceptable quantum efficiency (QE), strong blue light absorption, and small Stokes shifts [15]. *M*-α-SiAlON:Eu^2+^/Ce^3+^, with the structure derived from α-Si_3_N_4_ [46,47] and a general formula of M_m/v_Si_12–m–n_Al_m+n_O_n_N_16–n_, has been a prominent member of the nitride phosphors family [48–71]. M with a valence of v is typically alkaline earth or alkali metals such as Sr, Ca, and Li, which can be partially substituted by a slight amount of activator ions such as Eu^2+^ or Ce^3+^. They are made of corner-sharing (Si/Al)(N/O)_4_ tetrahedra, where Si – N bonds are partially substituted by Al – N and Al – O bonds. The M and rare-earth cations can be accommodated in the interstices of such a 3D network. Van Krevel et al. reported Eu-, Tb- and Ce-doped α-SiAlON phosphors [51]. Ce- and Eu-doped Ca–α-SiAlON exhibited bright long-wavelength luminescence, with maxima at 515–540 and 560–580 nm for Ce and Eu, respectively. These materials demonstrated high quantum efficiency and high absorption for 365- and 254-nm excitation. Xie et al. synthesized Ca-α-SiAlON:Ce^3+^, showing a broad emission spectra centered at 500–518 nm under near-UV excitation [55]. Xie et al. also reported yellow-emitting Ca-α-SiAlON:Eu^2+^ [68] and green-yellow-emitting Li-α-SiAlON:Eu^2+^ [69], and suggested their use in wLEDs when coupled to blue LED chips. However, it is hard to synthesize a single-phase Sr-based α-SiAlON:Eu^2+^ due to the large size of Sr^2+^, which destabilizes the α-SiAlON structure [48].

In 2010, Shioi et al. successfully addressed the challenge of preparing Sr-α-SiAlON:Eu^2+^ by using small values of m (0.7–0.8) and n (0–0.05), and obtained phase-pure yellow-emitting Sr_0.375_Al_0.77_Si_11.25_N_15.98_O_0.02_:2%Eu^2+^ at 2000°C under 1 MPa N_2_ pressure [72,73]. In 2017, Yoshimura and Yamane synthesized a new yellow-emitting Sr-α-SiAlON:Eu^2+^ single crystal (Sr_0.31_Al_0.62_Si_11.38_N_16_:1%Eu^2+^, λ_em_ = 583 nm under 400 nm excitation) with m = 0.62 and n = 0, the composition of which is close to the one reported by Shioi et al. [74] Further, they reported another yellow-emitting single crystal phosphor (Sr_3_Al_6_Si_24_N_40_:1%Eu^2+^, λ_em_ = 584 nm under 400 nm excitation), which is a novel polytypoid of α-SiAlON with space group of *P*$\overset{ˉ}{6}$ (No. 174) and lattice parameters of *a* = 7.948 Å and *c* = 14.394 Å. Its structure corresponds to the general formula of *M*-α-SiAlON (Sr_1.2_Al_2.4_Si_9.6_N_16_; *Z* = 0.4) with m = 2.4 and n = 0 [74]. The Sr content in this polytypoid structure exceeds the range reported by Shioi et al. by more than 200% [72,73].

In this work, we report a single-phase ‘Sr-rich’ Sr_3_Si_24_Al_6_N_40_:Eu^2+^ phosphor powder having a unique polytypoid α-SiAlON structure with three distinctive Sr/Eu luminescence sites, synthesized using a solid-state remixing-reannealing process. The effects of the Eu^2+^ concentration on the PL emission and excitation (PLE) spectra, QE, absorptance, and decay characteristics are investigated and discussed. A white LED is fabricated by combining a powder mixture of the title orange-yellow-emitting Sr_2.85_Eu_0.15_Al_6_Si_24_N_40_, commercial green-emitting SrSi_2_O_2_N_2_:Eu^2+^, and red-emitting Ca_0.9_Sr_0.1_AlSiN_3_:Eu^2+^ phosphors with a blue LED chip (455 nm).

## 2. Experimental procedure

### 2.1. Single-phase synthesis of polytypoid α-SiAlON Sr_3_Si_24_Al_6_N_40_:Eu^2+^ powders

α-Si_3_N_4_ (SN-E10, Ube Industries Ltd., Japan), Sr_3_N_2_ (Kojundo Chemical Laboratory Co. Ltd., Japan), SrSi_2_ (99%, Kojyundo Chemical Laboratory Co. Ltd., Japan), AlN (E-grade, L-101, Tokuyama Chemical Co. Ltd., Japan), and EuN powders (Materiaon 99.9%) were used as starting materials to prepare powder mixtures with varying compositions. The powders were carefully weighed and mixed using a Si_3_N_4_ mortar and pestle in a glove box (MBRAUN Unilab, MBRAUN GmbH, Germany) filled with nitrogen, where H_2_O and O_2_ contents were less than 1 ppm. About 2 g of each powder mixture was loaded into a BN crucible, and then fired in a gas-pressure furnace with a graphite heater (FVPHR-R-10, FRET-40, Fujidempa Kogyo Co. Ltd., Japan) at temperatures ranging from 1900 to 2050°C for 2 hours under 0.92 MPa N_2_ pressure. The fired powder mixtures were pulverized using a Si_3_N_4_ mortar and pestle, and finally used for further characterizations.

The compositions of powder mixtures were determined based on the formula Sr_1+x-y(1+x)_Eu_y(1+x)_Si_28-2x_Al_2 + 2x_N_40_:Eu^2+^, where x ranges from 1.8 to 2.2. In order to find the optimal x value, the Eu concentration was maintained at 5% (y = 0.05) of the total Sr and Eu content (1+x) for all compositions. Then, single-phase phosphor powders with varying Eu concentrations (y = 0.006 to 0.15) were synthesized at the fixed optimal x value.

### 2.2. Structural analyses

X-ray diffraction (XRD) patterns were obtained at ambient conditions using a MiniFlex 600 powder X-ray diffractometer (Rigaku, Japan) with Cu Kα radiation (λ = 1.54 Å) and a high-speed detector. The scanning speed was 3° min^−1^ with a 0.02 step, and the voltage and current were set to 40 kV and 15 mA, respectively. The scanning speed was determined based on the generally acceptable intensity required for the Rietveld refinement process (10,000 counts). The lattice constants were calculated by the Lattice Parameter Refinement module of the PDXL 2.0 software suite (Rigaku Corporation, Japan) using an external standard reference sample. High-quality XRD patterns (scanning speed of 1° min^−1^) obtained from the powders carefully leveled on a non-diffractive single-crystal silicon sample holder were used.

The morphology of phosphor particles was evaluated using a field-emission scanning electron microscope (FE-SEM) (JSM-7001F, JEOL, Japan) operated at 15 kV and a digital optical microscope with a 400 nm UV light source. The later was also used to identify phosphor impurity particles of different colors within the powders.

### 2.3. Chemical analysis

The concentrations of Sr, Eu, Al, and Si in different phosphor powders were measured using inductively coupled plasma optical emission spectrometry (ICP-OES) (SPS3520UV-DD, Hitachi High-Tech Science Co., Japan) with standard solutions from Kanto Chemical Co., Inc. The concentrations of nitrogen and oxygen were determined through the use of inert gas fusion techniques (TC-436AR, LECO Co., Japan). The analysis of nitrogen was carried out using the inert gas fusion-thermal conductivity method (JIS R 1603–8.3) with the Si_3_N_4_ standard qualified by the Ceramic Society of Japan. Oxygen was analyzed using the inert gas fusion-infrared absorption method (JIS R 1603–10.2) with the Y_2_O_3_ (99.999%) standard made by SPEX Certiprep, Inc. Two measurements were performed for each powder, and the mean values were reported.

X-ray photoelectron spectroscopy (XPS) was performed by a PHI Quantera SXM spectrometer (ULVAC-PHI, Inc., Japan) with monochromatic X-ray (Al Kα) excitation set at 100 W (20 kV, 5 mA) and a takeoff angle of 45 deg. Survey spectra were recorded with pass energy of 280 eV and energy step of 0.5 eV. Multiplex spectra were obtained with pass energy of 55 eV and energy step of 0.1 eV. For the Eu 3d high-resolution spectrum, pass energy of 112 eV and energy step of 0.1 eV were used. The energies were calibrated using C 1s peak at 285.0 eV.

### 2.4. Single-particle chemical analysis

The electron probe microanalyzer (EPMA) (JEOL JXA-8500F, Japan) was used for quantitative chemical analysis and elemental mapping of each phosphor particle. Quantitative analysis was performed using an accelerating voltage of 15 kV, probe current of 20 nA, and 30 s measurement time. Elemental mapping was conducted with an accelerating voltage of 15 kV, probe current of 50 nA, 25 ms measurement time, and step width of 0.25 µm. A cross-sectional sample was prepared by embedding 100 mg of Sr_2.85_Eu_0.15_Si_24_Al_6_N_40_ (5% Eu^2+^) powder in an epoxy resin. The hardened resin was then polished to create a flat surface that exposed the particles’ cross-sectional surfaces.

### 2.5. PL characterizations

A steady-state fluorescence spectrometer (F-4500, Hitachi Ltd., Japan) equipped with a 200 W Xe lamp as the excitation source was used to record the PL and PLE spectra of phosphor powders at ambient conditions. The spectra were also corrected for the instrumental responses.

The spectra used for the measurements of the absorption capacity and quantum efficiency (QE) were obtained using an intensified multichannel spectrometer (QE2100, Otsuka Electronics, Japan) coupled with an integrating sphere. For calibration, we used the reflection spectrum of the Spectralon diffusive white standard (BaSO_4_). The internal (η_i_) and external (η_0_) QE values were calculated according to the following equations [75]:

(1) $$\eta_{0}=\frac{\int \lambda .P\left(\lambda \right)\mathrm{d\lambda}}{\int \lambda .E\left(\lambda \right)\mathrm{d\lambda}}$$

(2) $$\eta_{i}=\frac{\int \lambda .P\left(\lambda \right)\mathrm{d\lambda}}{\int \lambda \left[E\left(\lambda \right)-R\left(\lambda \right)\right]d\lambda }$$

The intensity per unit wavelength in the reflectance, excitation, and emission spectra are represented by *R*(λ), *E*(λ), and *P*(λ), respectively.

The luminescence decay curves were acquired with a time-correlated single photon counter (TemPro, Horiba Jobin Yvon, Japan), utilizing a 370 nm pulsed NanoLED with a pulse duration full width at half-maximum (FWHM) of approximately 1 ns. The measurements were performed at ambient conditions. Equation (3) was used to calculate the average lifetime values for the double and triple exponential fitted curves. The values of *T* and *B* represent the lifetime and intensities contributed by each exponential component, respectively [76].

(3) $$\tau =T_{1}\left[B_{1}/\left(B_{1}+B_{2}+B_{3}\right)\right]\;+T_{2}\left[B_{2}/\left(B_{1}+B_{2}+B_{3}\right)\right]\;+T_{3}\left[B_{3}/\left(B_{1}+B_{2}+B_{3}\right)\right]$$

The temperature-dependent PL spectra under 400 nm excitation were recorded at ambient conditions in both heating and cooling cycles using an intensified multichannel spectrometer (MCPD-7000, Otsuka Electronics, Japan). The phosphor powders were gradually heated to 300°C at a heating rate of 100°C min^−1^ in a 50°C intervals and held at each temperature for 5 min before measurements.

### 2.6. Fabrication of pc-wLED

We fabricated white LEDs by pumping a phosphor mixture of the orange-yellow-emitting Sr_2.85_Eu_0.15_Al_6_Si_24_N_40_, green-emitting SrSi_2_O_2_N_2_:Eu^2+^, and red-emitting Ca_0.9_Sr_0.1_AlSiN_3_:Eu^2+^ powders with a blue LED chip (455 nm). The optical properties of white LEDs were measured with an integrated sphere Spectroradiometer system (LHS-1000, Everfine Co., China) while operating at a voltage of 3.756 V and a bias current of 300 mA.

## 3. Results and discussion

**Figure 1.**
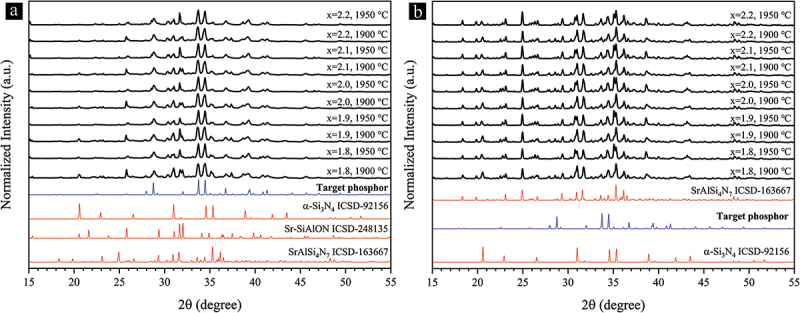
XRD patterns showing the general effect of different Sr^2+^ sources on the phase purity of the Sr_3_Si_24_Al_6_N_40_:Eu^2+^ phosphor powders synthesized at 1900°C and 1950°C: (a) Sr_3_N_2_ and (b) SrSi_2_ were used as the Sr^2+^ sources.

To determine the optimal route for synthesizing single-phase Sr_3_Si_24_Al_6_N_40_:Eu^2+^ phosphor powders, we initially examined the effect of two different Sr^2+^ sources on the phase purity of the synthesized powders. The synthesis of nitride phosphors demands no oxidation of the starting materials before firing. Thus, Sr_3_N_2_ and SrSi_2_ were used to prepare different powder mixtures according to the formula Sr_1+x-y(1+x)_Eu_y(1+x)_Si_28-2x_Al_2 + 2x_N_40_:Eu^2+^, with the Eu^2+^ doping set to 5% (y = 0.05) and x in the range of 1.8–2.2. SrSi_2_ has much better oxidation resistance than Sr_3_N_2_ or pure Sr [73]. Figure 1 displays the XRD patterns of the mixtures fired at 1900 and 1950°C under 0.92 MPa N_2_. The ICSD XRD patterns of the major phases and the simulated target phase (Sr_2.97_Eu_0.03_Si_24_Al_6_N_40_) [74] are also presented. The target phosphor is the main phase in the mixtures prepared with Sr_3_N_2_, while it is negligible in those prepared with SrSi_2_. As a result, we used all-nitride starting powders in this work.

**Figure 2.**
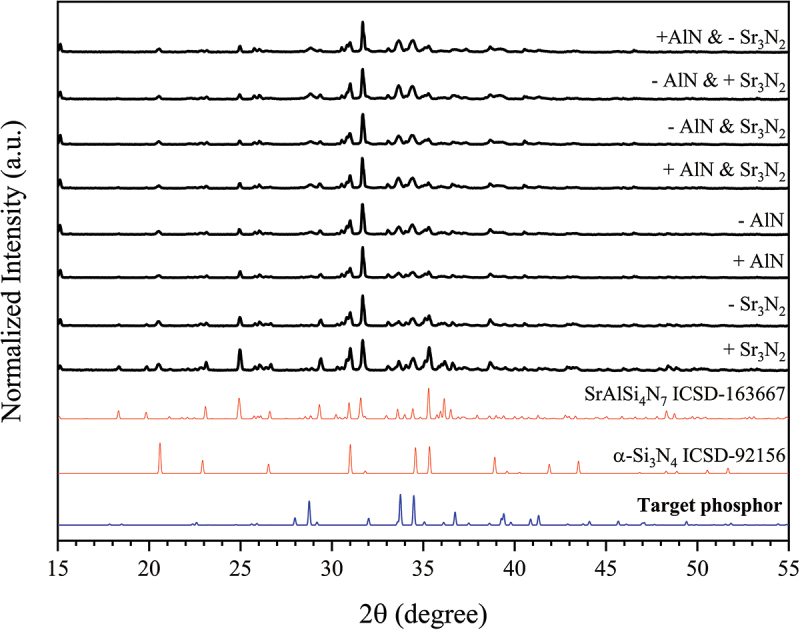
XRD patterns showing the effect of non-stoichiometric compositions on the phase purity of the Sr_3_Si_24_Al_6_N_40_:Eu^2+^ phosphor powders synthesized at 1900°C. The + and – signs indicate the relative increase or decrease in the amount of AlN and Sr_3_N_2_ with respect to the stoichiometric composition (x = 2.2). For example, the XRD pattern labeled ‘+ Sr_3_N_2_’ corresponds to the mixture with an increased Sr_3_N_2_ from the stoichiometric value (1.0%), while AlN, α-Si_3_N_4_, and EuN are at their stoichiometric values.

The effect of non-stoichiometric compositions on the phase purity of the synthesized powders was also investigated to address possible evaporations of the starting materials during firing. For this purpose, eight different mixtures with varying contents of Sr_3_N_2_ and AlN were prepared and treated at 1900°C. The increase or decrease in the amount of Sr_3_N_2_ and AlN relative to the stoichiometric composition (x = 2.2) was set at approximately 1.0% of the total weight of the powder mixtures (2 g). The x value of 2.2 was chosen because it resulted in the lowest α-Si_3_N_4_ phase after firing. As demonstrated in Figure 2, this non-stoichiometric composition design strategy leads to a major phase of SrAlSi_4_N_7_ phosphor (ICSD-163667) as well as a considerable amount of α-Si_3_N_4_. The existence of SrAlSi_4_N_7_, α-Si_3_N_4_ or other (oxy)nitrides [73,74,77–82] indicates that it is essential to use a precise stoichiometric composition to obtain single-phase Sr_3_Si_24_Al_6_N_40_:Eu^2+^ powders.

**Figure 3.**
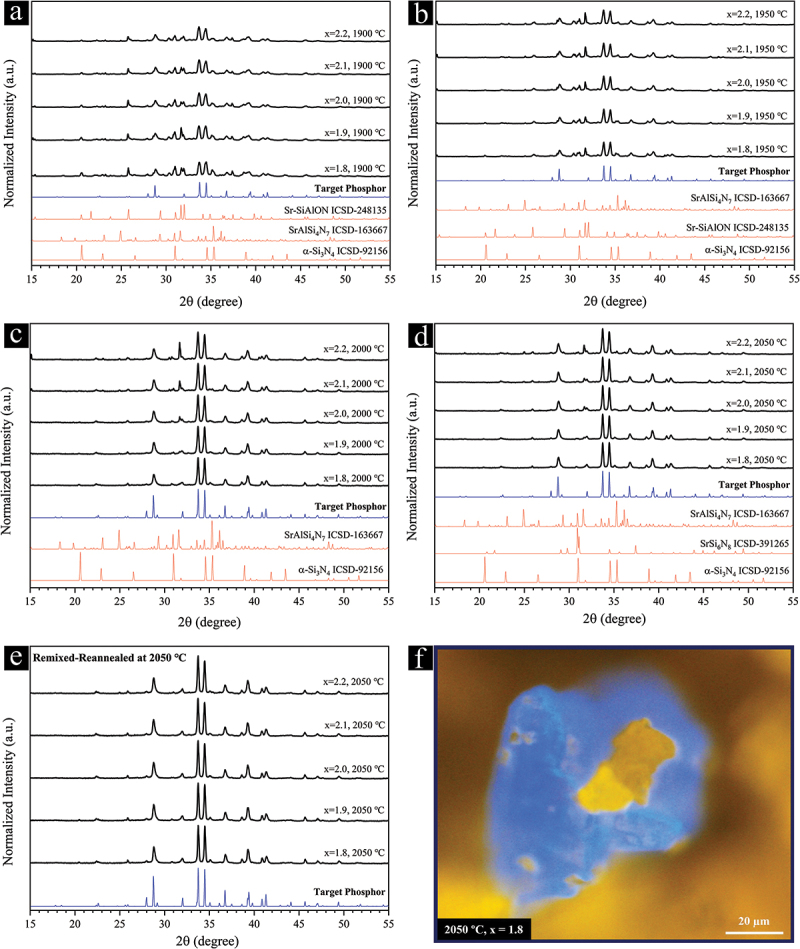
XRD patterns showing the effect of stoichiometric compositions on the phase purity of the Sr_3_Si_24_Al_6_N_40_:Eu^2+^ phosphor powders synthesized at (a) 1900°C, (b) 1950°C, (c) 2000°C, and (d) 2050°C. (e) XRD pattern of the 2050°C-treated powders after remixing and subsequent annealing at 2050°C for 2 h. (f) UV-illuminated optical microscopy image of a blue-emitting particle possibly synthesized between 2000°C and 2050°C (x = 1.8), which is decomposing into the yellow-emitting Sr_3_Si_24_Al_6_N_40_:Eu^2+^ phase from the inside.

**Figure 4.**
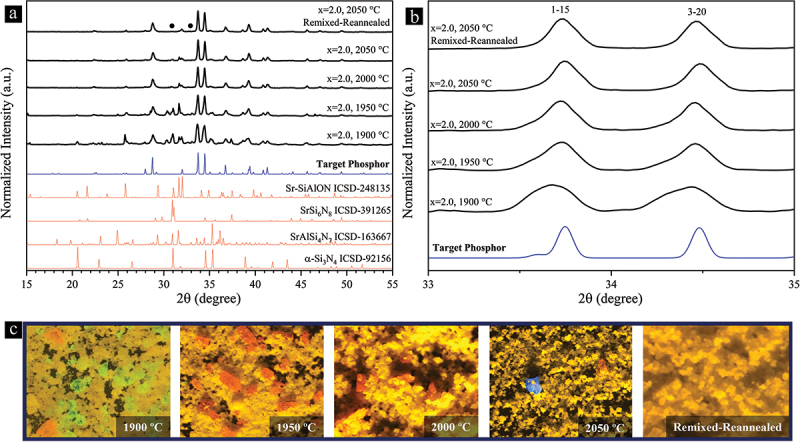
(a) XRD patterns showing the effect of temperature on the phase purity of the synthesized Sr_3_Si_24_Al_6_N_40_:Eu^2+^ phosphor powders with the optimal composition (x = 2). The uppermost pattern in (a) includes two small, solid dots that correspond to tiny peaks of an unknown phase or phases. (b) The evolution of the two strongest peaks of our target phosphor phase with the change in temperature. (c) UV-illuminated optical microscopy images of the synthesized powders revealing the impurity phosphor particles (blue-, green-, and red-emitting) within the orange-yellow-emitting Sr_3_Si_24_Al_6_N_40_:Eu^2+^ matrix particles. No phosphor impurities are present in the remixed-reannealed powder.

The impact of stoichiometric compositions, all-nitride starting powders, and treatment temperature on the phase purity of the synthesized phosphor powder was investigated, and the results are presented in Figures 3 and 4. Irrespective of the x value and temperature, the major phase is the target Sr_3_Si_24_Al_6_N_40_:Eu^2+^ phosphor. At 1900°C, the main impurities are detected as SrAlSi_4_N_7_:Eu^2+^ [83], Sr-SiAlON (Sr_3_Si_13_Al_3_O_2_N_21_:Eu^2+^) [84], and α-Si_3_N_4_, and their amounts decrease as x increases. Similar phases can be detected at 1950°C, and their amounts seem to be rather independent of x. At 2000°C, the Sr-SiAlON phase disappears, leaving behind smaller amounts of SrAlSi_4_N_7_ as the main impurity, which increases with x. Perhaps the weak reducing atmosphere of the graphite-heater furnace, which enables the reduction of Eu^3+^ into Eu^2+^, was intensified at 2000°C, leading to the decomposition of the green-emitting Sr-SiAlON phase. The impurity level is significantly reduced at 2050°C with the minor detectable phosphor phases being the red-emitting SrAlSi_4_N_7_:Eu^2+^ and possibly blue-emitting SrSi_6_N_8_:Eu^2+^. For the latter, phase confirmation is difficult. A prime example of such decomposition is demonstrated in Figure 3f, where a blue-emitting phosphor particle decomposes into our target orange-yellow-emitting phase from the inside at 2050°C. Such few blue-emitting particles may be stable between 2000 and 2050°C, as they are not observed at 2000°C and begin to decompose at 2050°C. This blue-emitting particle cannot be SrSi_6_N_8_:Eu^2+^ as it lacks the Al required for the crystallization of the Sr_3_Si_24_Al_6_N_40_:Eu^2+^ phase. The optimal value of x is determined to be 2 due to comparatively negligible amounts of α-Si_3_N_4_ and SrAlSi_4_N_7_:Eu^2+^ impurities at 2050°C (Figure 3d). Ultimately, remixing the powders obtained at 2050°C, followed by subsequent reannealing under similar conditions, leads to the perfect elimination of all phosphor impurities at x values of 1.8 to 2 (Figure 3e). In contrast to the powders with x = 1.8 and 1.9, no α-Si_3_N_4_ can also be detected at x = 2. At x = 2.1, blue-emitting particles are barely visible with an optical microscope under UV excitation. Negligible red- and blue-emitting phosphors can also be detected at x = 2.2. Remixing the powders might have brought the unreacted, isolated starting materials and impurity phosphor phases into contact with the rest of the particles, leading to their complete reaction and elimination during the subsequent reannealing at 2050°C.

Figure 4 clearly demonstrates the significant impact of temperature and remixing-reannealing process on the single-phase synthesis of the Sr_3_Si_24_Al_6_N_40_:Eu^2+^ powders, even with the optimal composition (x = 2). The evolution of the two most prominent peaks of our target phosphor phase reflects the elimination of impurity phases with overlapping peaks as the temperature increases (Figure 4b). UV-illuminated optical microscopy images of the synthesized powders reveal impurity phosphor particles detected at each temperature. Consistent with the XRD analysis, the detected green- (1900 and 1950°C) and red-emitting (1900 to 2050°C) particles correspond to a Sr-SiAlON phase (Sr_3_Si_13_Al_3_O_2_N_21_:Eu^2+^) [84] and SrAlSi_4_N_7_:Eu^2+^ [83], respectively.

**Figure 5.**
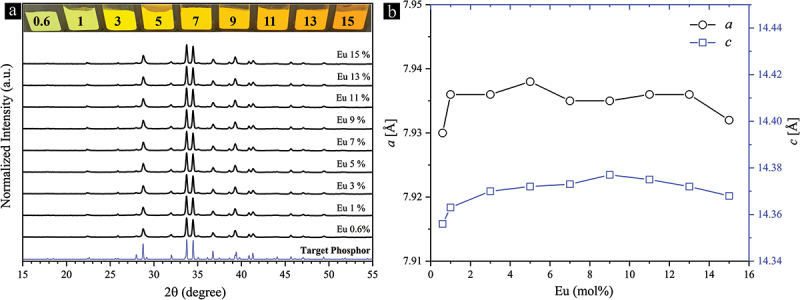
(a) XRD patterns showing the effect of Eu^2+^ concentration on the phase purity of the Sr_3_Si_24_Al_6_N_40_:Eu^2+^ phosphor powders synthesized using the optimal stoichiometric composition (x = 2) and the remixing-reannealing process at 2050°C. The inset displays the body color of the single-phase phosphor powders. (b) Lattice parameters versus Eu^2+^ concentration.

We were able to synthesize single-phase Sr_3_Si_24_Al_6_N_40_:Eu^2+^ phosphor powders with varying Eu^2+^ concentrations using the optimal stoichiometric composition (x = 2) and the remixing-reannealing approach (at 2050°C). The phase purity was thoroughly assessed through a comprehensive suite of analytical techniques, including XRD, UV microscopy, XPS, ICP-OES, and inert gas fusion techniques. The XRD patterns, body color, and calculated lattice constants of these powders are presented in Figure 5. As the Eu^2+^ concentration increases, the XRD patterns remain totally unchanged, despite the change in body color from light green to orange. Perhaps Eu^2+^, with a similar ionic size and oxidation state to Sr^2+^ [85,86] is easily accommodated in the three large Sr^2+^-centered cages of the structure without causing any strains or instability in the structure [74]. The parameter *a* increases with Eu^2+^ doping, reaching a maximum expansion of 0.008 Å at 5%. Further doping decreases this parameter. However, when excluding the 0.6% and 15% Eu^2+^-doped powders, parameter *a* is almost independent of the doping level within the 1–13% Eu^2+^ range (maximum ∆*a* = 0.003 Å). Parameter *c* also increases with Eu^2+^ doping, reaching a maximum expansion of 0.021 Å at 9%. The decrease in *c* from 9 to 15% Eu^2+^ doping is 0.009 Å. Comparing the lattice constants of the 1% Eu^2+^-doped powders to those of the 1% Eu^2+^-doped single crystals Sr_2.97_Eu_0.03_Si_24_Al_6_N_40_ [74], the powder shows reductions of 0.012 Å and 0.031 Å in the parameters *a* and *c*, respectively. Since a full Rietveld refinement could not be applied to our patterns due to their extreme complexity, the absolute lattice parameter values reported here might include possible errors. Nevertheless, they are reliable for comparison purposes, as they were obtained using the same XRD setup and precisely analyzed using the same Lattice Parameter Refinement module of the PDXL 2.0 software suite (Rigaku Corporation, Japan).

**Figure 6.**
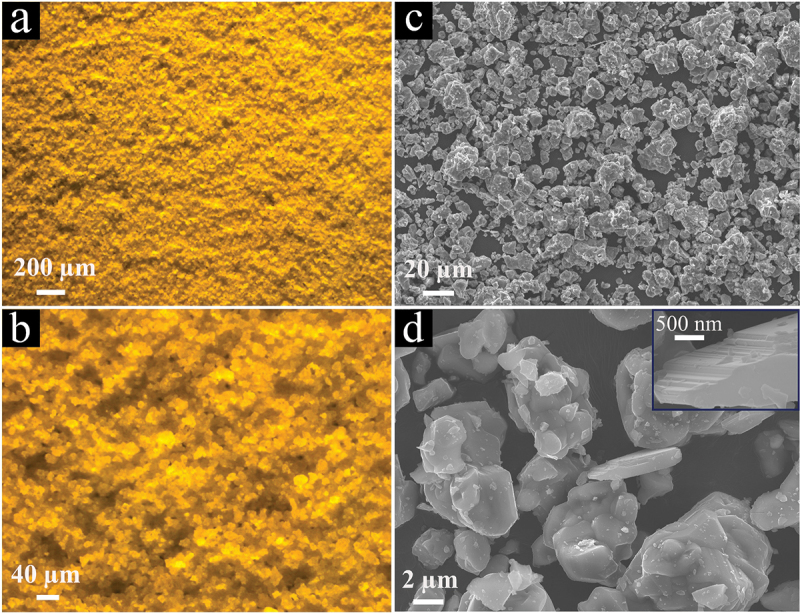
Typical UV-illuminated optical microscopy (a,b) and SEM images (c,d) of the single-phase 5% Eu^2+^-doped Sr_3_Si_24_Al_6_N_40_ phosphor powder.

Figure 6 displays the typical UV-illuminated optical and SEM images of the 5% Eu^2+^-doped powders, confirming their phase purity and providing insights into the morphology of the particles. Optical images 6a and 6b are typical examples of the many images observed to detect any phosphor impurities within this powder. The absence of other phosphor particles in various samples is fully consistent with the XRD analysis. The particles have a rather uniform size distribution, with irregular shapes different from the block-shaped single crystals [74]. Some particles appear to be polycrystals.

**Figure 7.**
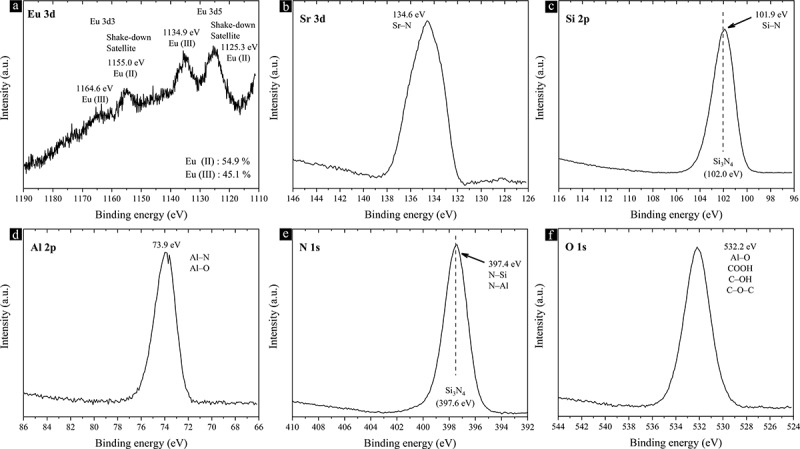
High-resolution XPS spectra of the single-phase 5% Eu^2+^-doped Sr_3_Si_24_Al_6_N_40_ phosphor powder.

The results of the quantitative bulk chemical analysis of the single-phase phosphor powders doped with 0.6%, 3%, and 5% Eu^2+^ are summarized in Table 1. The measured values are consistent with the nominal composition of each powder. Minor oxygen impurities may have originated from the surface oxidation of the powders during grinding, storage, and analysis performed under ambient conditions. The amount of oxygen is nearly identical in all the analyzed powders, as they have nearly the same particle morphology and surface area. XPS measurements were conducted on the 5% Eu^2+^-doped powders to gain insights into their surface composition and the chemical states of each element. The surface composition derived from the survey spectra is listed in Table 2. The large quantity of oxygen confirms surface oxidation of the phosphor particles and supports the quantitative bulk chemical analysis conducted by the ICP-OES method and the inert gas fusion analyses. The surface composition is in good agreement with the measured and nominal bulk compositions when oxygen is excluded from the calculation. High-resolution core-level spectra are shown in Figure 7. The presence of Eu^3+^ (1135.8 eV in Eu_2_O_3_) has been confirmed based on the Eu 3d spectra, while Eu^2+^ accounts for 54.9% [87]. The most intense binding energy of 134.6 eV in the Sr 3d spectrum corresponds to the Sr – N bond. However, the peak broadening may be due to the presence of Sr – O bonds with energies ranging from 133.4 eV to 135.5 eV. The Si 2p spectrum confirms the presence of only Si – N bonds, similar to Si_3_N_4_ (102.0 eV), and no Si – O bonds. Meanwhile, the Al 2p spectrum indicates the presence of both Al – N and Al – O bonds. Thus, at Si/Al sites, only Al atoms appear to be oxidized on the surface. The N 1s spectrum verifies the presence of N – Si and N – Al bonds. The broad O 1s spectrum suggests the presence of carbon-based impurities (COOH, COH, and COC) on the surface, which could explain the high levels of oxygen and carbon detected in the survey spectra.

**Table 1.** Chemical compositions of the single-phase 0.6, 3, and 5% Eu^2+^-doped phosphor powders, including the nominal compositions as references. The ICP-OES method (for Sr, Eu, Al, and Si) and the inert gas fusion analyses (for N and O) were used to determine the composition.

Compositions (at.%)	Sr	Eu	Si	Al	N	O
5% Eu^2+^ - Nominal	3.904	0.205	32.877	8.219	54.794	0
**5% Eu**^**2+**^**- Measured**	**3.768**	**0.217**	**32.798**	**8.556**	**54.227**	**0.433**
3% Eu^2+^ - Nominal	3.986	0.123	32.877	8.219	54.795	0
**3% Eu**^**2+**^**- Measured**	**3.824**	**0.136**	**32.766**	**8.505**	**54.068**	**0.573**
0.6% Eu^2+^ - Nominal	4.084	0.025	32.877	8.219	54.795	0
**0.6% Eu**^**2+**^**- Measured**	**3.940**	**0.024**	**32.564**	**8.559**	**54.359**	**0.428**

**Table 2.** Surface chemical composition of the single-phase 5% Eu^2+^-doped phosphor powder derived from the XPS survey spectra (in at.%). The first row displays the relative surface compositions considering all the detected elements, including impurities and oxygen in the calculation, while the second row considers only the elements of the designed phosphor powder.

C 1s	N 1s	O 1s	Al 2p	Si 2p	Cl 2p	Ca 2p	Fe 2p	Zn 2p	Sr 3d	Eu 3d5
30.3	25.1	21.8	3.8	15.7	0.3	0.2	0.5	0.1	2.0	0.2
0	53.63	0	8.12	33.54	0	0	0	0	4.27	0.42

**Figure 8.**
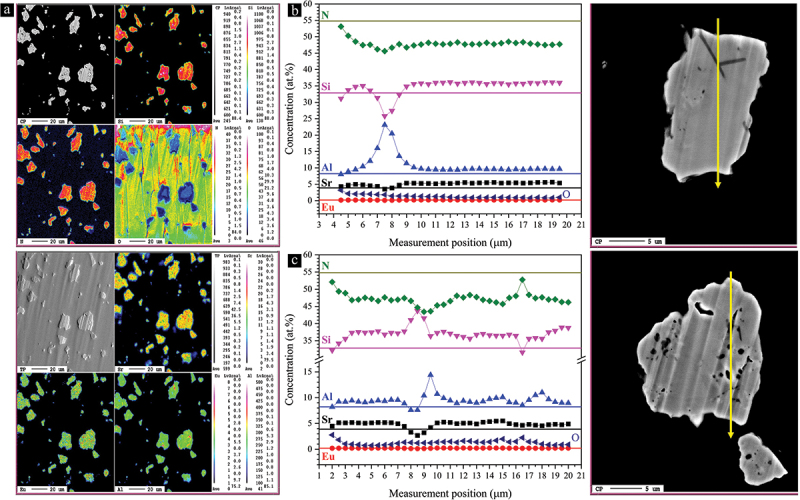
(a) EPMA elemental mapping of some polished phosphor particles of the single-phase 5% Eu^2+^-doped Sr_3_Si_24_Al_6_N_40_ phosphor powder. (b,c) quantitative EMPA line analysis of two polished particles with their corresponding SEM images. The colored straight lines correspond to the starting stoichiometric composition.

Single-particle chemical analysis was also performed on the polished cross-sections of some phosphor particles using EPMA, as shown in Figure 8. The quantitative line analysis scanning the particles verifies their uniform compositions, which are consistent with the nominal value, particularly for the heavier elements. Elemental mapping also confirms the uniformity of the composition throughout the particles. Oxygen is negligible, which is consistent with the results of the inert gas fusion analyses and XPS. The SEM images reveal the presence of small pores within the particles, which could potentially affect their PL properties. Such pores are responsible for the points with deviated compositions in Figure 8b,c.

**Figure 9.**
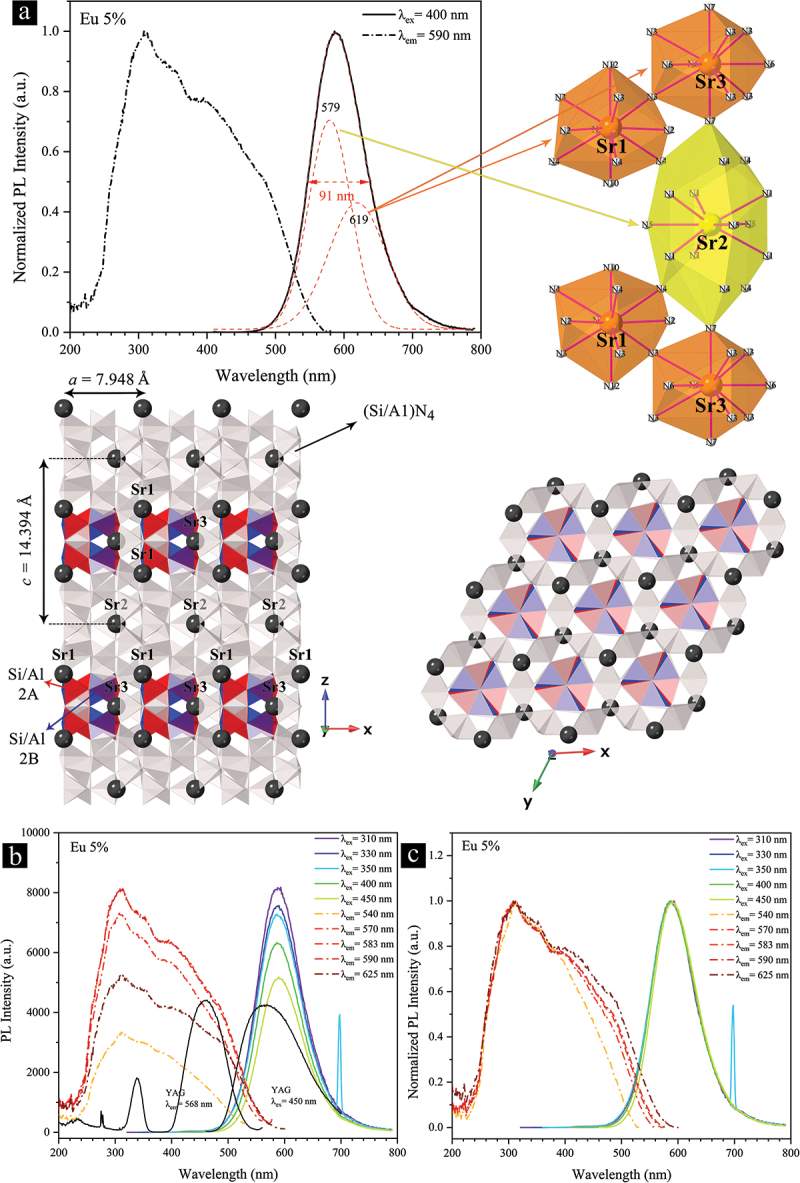
(a) Normalized PL and PLE spectra of the single-phase 5% Eu^2+^-doped Sr_3_Si_24_Al_6_N_40_ phosphor powder recorded in ambient conditions. The illustrated crystal structure of the phosphor highlights three distinct Sr/Eu sites with different surrounding N-atom arrangements and polyhedral volume, and a distinct Si/Al split site (red/blue) among five other similar Si/Al sites. This illustration was created using the CrystalMaker software and the single crystal structure data provided by Yoshimura and Yamane [74]. (b) PL and PLE spectra monitored at different wavelengths. (c) Normalized version of (b).

Figure 9a shows the PL and PLE spectra of the single-phase 5% Eu^2+^-doped powders and illustrations of the corresponding crystal structure. The PL spectrum, recorded under 400 nm excitation, ranges from approximately 460 nm to 780 nm, with the emission maximum at 590 nm. This broad emission band corresponds to the electric dipole-allowed 4*f*^7^-4*f*^6^ 5*d*^1^ transitions of Eu^2+^ from the lowest level of excited state (5*d*) to the ground state (4*f*) [13,88,89], and is slightly broader than the PL band of the single crystal [74]. The PLE spectrum, monitored at 590 nm, is broad, covering from approximately 230 nm to 570 nm, with a dominant peak and several shoulders at approximately 310 nm, 355 nm, 400 nm, and 485 nm. The most intense peak at ~310 nm can be attributed to the light absorption of the novel polytypoid Sr-α-SiAlON host. In the yellow-emitting Ca-α-SiAlON:Eu^2+^ phosphor, the PLE peak below 300 nm was also regarded as the characteristic of the host material [68]. The remaining shoulder peaks can be explained by the allowed 4*f*^7^→4*f*^6^ 5*d*^1^ transitions of Eu^2+^ in the α-SiAlON hosts [72–74,86].

The broad PL band can be readily deconvoluted into two distinct Gaussian peaks centered at 579 nm and 619 nm, which reflects the existence of different Eu^2+^ luminescence centers. As illustrated in Figure 9a, the Sr_3_Si_24_Al_6_N_40_:Eu^2+^ crystal has three Sr/Eu sites with different nitrogen coordination environments. The Sr1 and Sr3 sites have both 11-fold coordination environments with average Sr – N lengths of 2.849 Å and 2.851 Å, respectively. These interatomic distances are close to the average Sr – N distances within the 11-fold coordination of Sr/Ca sites in α-SiAlON (2.810 Å for Sr site and 2.803 Å for Ca site) [73] and the 11-fold coordination of Sr site in the recently discovered Sr_0.31_Al_0.62_Si_11.38_N_16_ (2.806 Å) [74]. However, the Sr1 and Sr3 sites of the polytypoid α-SiAlON Sr_3_Si_24_Al_6_N_40_ crystal are located at different crystallographic sites (2*g* and 1*f* versus 2*b*) of a different crystal structure (*P*31*c* versus *P*$\overset{ˉ}{6}$), and their occupancies are almost twice (0.64 for Sr1 and 0.72 for Sr3). Therefore, they have unique coordination environments among the α-SiAlON structures. The Sr2 site, on the other hand, has a full occupancy and a nine-fold coordination environment within the Sr – N length of 2.976 Å with an average value of 2.939 Å. Furthermore, the Sr2 site is enclosed by a cage of 17 nitrogen atoms with a volume of 103.4 Å^3^, which is almost twice that of the Sr1 and Sr3 sites (~51.5 Å^3^). This large space could be responsible for stabilizing this novel polytypoid α-SiAlON structure with a record-breaking 4.1% Sr content (*m* = 2.4). As a result, the Sr1/Eu1 and Sr3/Eu3 sites could possess comparatively more covalent coordination environments than the Sr2/Eu2 site, which enables longer emissions through a greater centroid shift and stronger crystal field splitting of 5*d* levels of Eu^2+^ [90,91]. The Si/Al split sites highlighted in red and blue colors do not affect the coordination environments of the Sr/Eu sites, and instead could be responsible for the red-shifted PLE maximum peak (~310 nm) assigned to the host material.

The PL and PLE spectra monitored at different wavelengths and their normalized versions are demonstrated in Figures 9b,c. The low-energy portion of the PLE spectrum continuously expands as the monitored wavelength increases from 540 nm to 625 nm. Such dependence of the PLE spectrum on the monitored wavelength is generally due to the presence of multiple Eu^2+^ sites in the crystal [92]. The high-energy portion of the PLE spectrum, including the most intense peak at ~310 nm, is almost independent of the monitored emission wavelength. Meanwhile, the position and shape of the PL band are independent of the excitation wavelength, while its intensity increases with the excitation energy. The PL intensity of the 5% Eu^2+^-doped powders is almost twice that of YAG under 450 nm excitation, which is attributed to its broad excitation spectrum.

**Figure 10.**
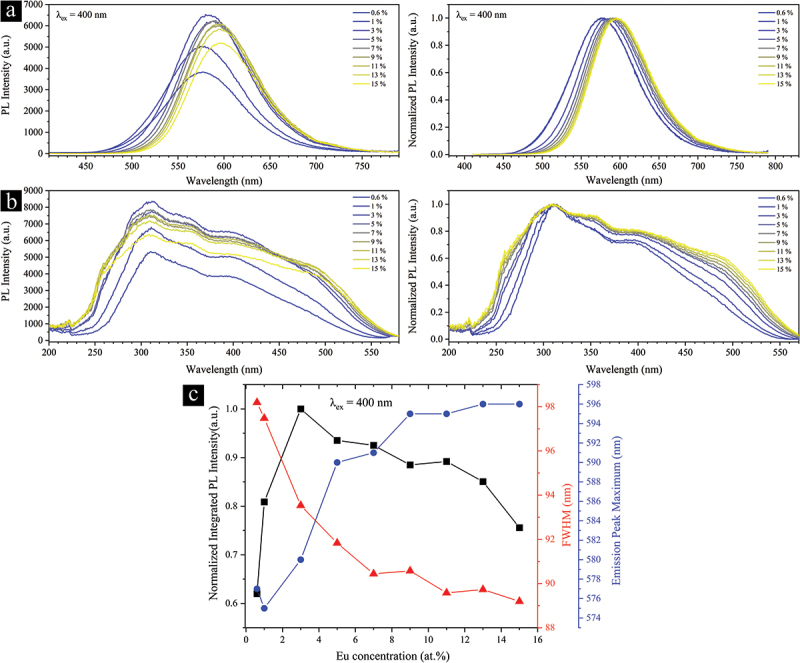
Eu^2+^ concentration-dependent (a) PL and (b) PLE spectra of the single-phase Eu^2+^-doped Sr_3_Si_24_Al_6_N_40_ phosphor powders recorded in ambient conditions. The excitation spectra were monitored at their corresponding peak emission wavelengths. (c) Dependence of the integrated PL intensity, FWHM values, and the emission peak maximum wavelength on the Eu^2+^ concentration.

The PL and PLE spectra are considerably influenced by the concentration of Eu^2+^, as demonstrated in Figure 10. As seen, the concentration quench occurs at 3% Eu^2+^, owing to the enhanced nonradiative energy transfer among Eu^2+^ ions [93]. It is a well-established fact that such energy transfer is inversely proportional to the nth power of their distance (*n* = 6, 8, or 10), which is directly influenced by their concentration. The PL band is also redshifted by ~19 nm (577 nm at 0.6% to 596 nm at 15%) and becomes narrower with increasing the Eu^2+^ concentration. The changes in the position and FWHM of the PL band reach a saturation level at 9% Eu^2+^. Particularly interesting given the narrowing of the PL band, the increase in Eu^2+^ has resulted in broadening and enhancement in both the high- and low-energy portions of the PLE spectrum (see normalized spectra in Figure 10b). This observation may be mainly attributed to the changes in the relative occupation level of the different Sr sites by the Eu^2+^ ions as the Eu^2+^ concentration increases. As illustrated in Figure 9a, the Sr2 site is fully occupied and located in a large cage with a volume of 103.4 Å^3^, which is more than twice that of the Sr1 and Sr3 sites with the site occupancy of 0.64 and 0.72, respectively. Thus, the preferential occupation of the Sr2 site by the Eu^2+^ ions may be relatively higher in low Eu^2+^ concentrations. As the amount of Eu^2+^ increases, it is forced to occupy the other two Sr sites, which have different coordination environments than the Sr2 site, as discussed earlier. In the absence of concentration quenching, having the Eu^2+^ ions located in multiple coordination environments can lead to the formation of more energy levels and a relative increase in the absorptance and emission intensity under a given excitation wavelength.

**Figure 11.**
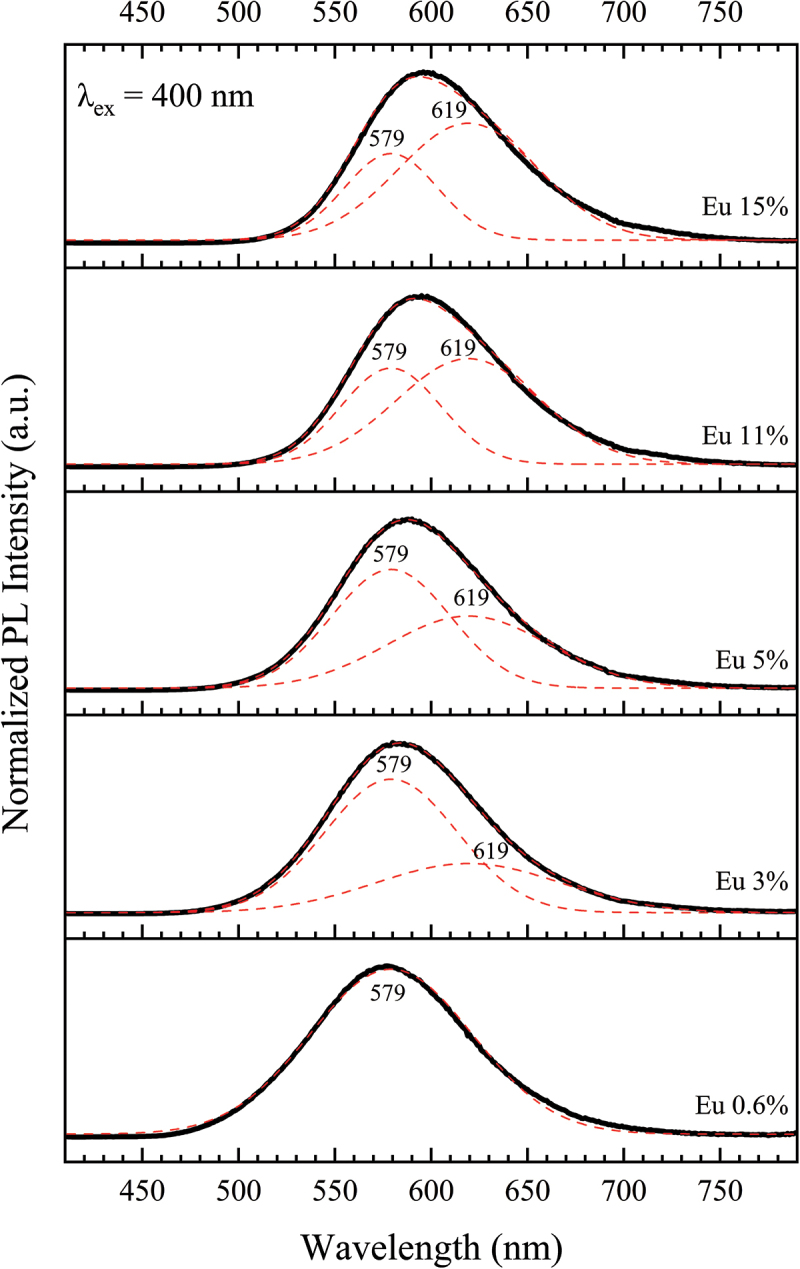
Normalized deconvoluted PL spectra of the single-phase Eu^2+^-doped Sr_3_Si_24_Al_6_N_40_ phosphor powders at different Eu^2+^ concentrations under 400 nm excitation.

The changes in the relative occupation degree of the Sr sites by the Eu^2+^ ions can also be observed in the PL spectra, as shown in Figure 11. The PL band of the 0.6% Eu^2+^-doped powders is well fitted by a single Gaussian peak centered at 579 nm. Under the simple assumption that the Eu^2+^ ions preferentially occupy only the Sr2 site, this peak can be regarded as a characteristic of the Sr2/Eu2 coordination environment. By fitting the PL spectra of the 3% Eu^2+^-doped powders using a fixed Gaussian peak centered at 579 nm, a peak centered at 619 nm is obtained, which can be considered the characteristic of the Sr1/Eu1 and Sr3/Eu3 coordination environments. The other powders are then well fitted using these two Gaussian peaks. Accordingly, a clear change in the relative contribution of these peaks or coordination environments in the formation of the PL spectrum can be observed. These analyses suggest that the low-energy Sr/Eu sites have a greater contribution to the overall emission and excitation characteristics as the concentration of Eu^2+^ increases, which also explains the red shift of the emission band by 19 nm in the Eu^2+^-concentrated powders. Another interesting point in the PLE spectra is the formation of a new shoulder at ~260 nm for powders with Eu^2+^ concentrations of 9% and above (Figure 10b). Further studies are necessary to understand its origin. Furthermore, the shoulder at 485 nm is absent or significantly attenuated in the 0.6% and 1% Eu^2+^-doped powders, which could be due to the lack of Eu^2+^ ions in the low-energy Sr1 and Sr3 sites.

**Figure 12.**
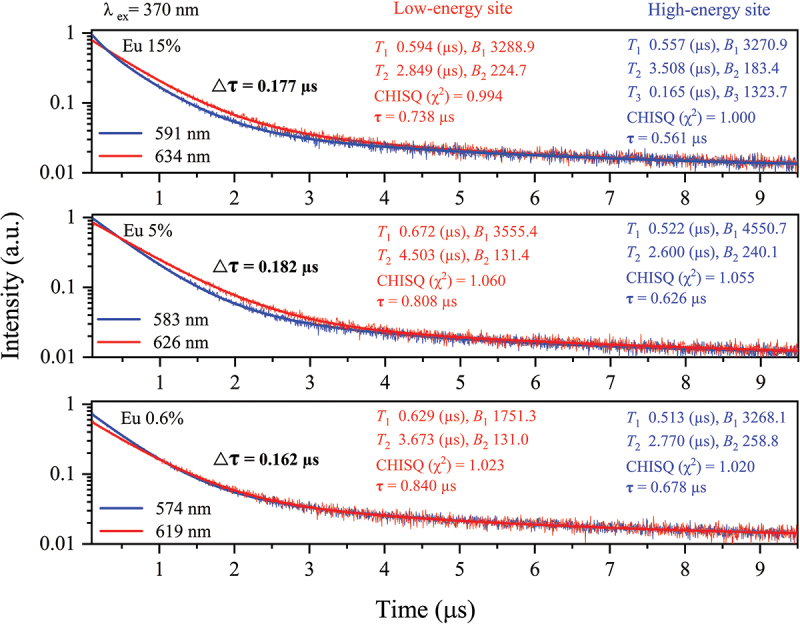
Luminescence decay curves of the single-phase 0.6%, 5%, and 15% Eu^2+^-doped Sr_3_Si_24_Al_6_N_40_ phosphor powders monitored at different wavelengths under 370 nm excitation. The exponential components of the fitted curves are characterized by *T* and *B* values, which are lifetime and contributed intensity, respectively.

Analysis of the luminescence decay curves can provide additional insights into the existence and lifetime of various Eu^2+^ luminescence centers in the Sr_3_Si_24_Al_6_N_40_:Eu^2+^ phosphors. The PL decay curves of three phosphor powders containing 0.6%, 5%, and 15% Eu^2+^ were monitored at different wavelengths around their maximum emission wavelengths. As displayed in Figure 12, all powders exhibit non-linear decay curves that can be readily fitted using two-exponential or three-exponential functions. The sole observation of a double exponential decay behavior does not necessarily indicate the existence of multiple Eu^2+^ luminescence sites. Similar double exponential decay behaviors were also observed in phosphors with single luminescence sites, possibly due to the existence of structural disorders and defects that affect the coordination environment [94,95]. However, our powders show different decay curves and lifetimes at various monitored wavelengths, which could be characteristic of a system with multiple luminescence centers and coordination environments [13,76]. It is widely accepted that the lifetime is mainly influenced by the local coordination environment through nonradiative relaxation processes [96]. In the 0.6 Eu^2+^-doped powders, the lifetime corresponding mainly to the low- and high-energy sites is calculated to be 0.840 µs and 0.678 µs, respectively, which falls within the radiative lifetime range of Eu^2+^ in various hosts [97]. The lifetime corresponding to both sites decreases as the Eu^2+^ increases, reaching 0.738 µs and 0.561 µs at 15%, respectively. In addition, regardless of their concentration, the luminescence of Eu^2+^ ions located in the high-energy Sr2 sites decays slightly faster than those in the low-energy Sr1 and Sr3 sites. These behaviors can be explained by the nonradiative energy transfer among Eu^2+^ ions.

**Figure 13.**
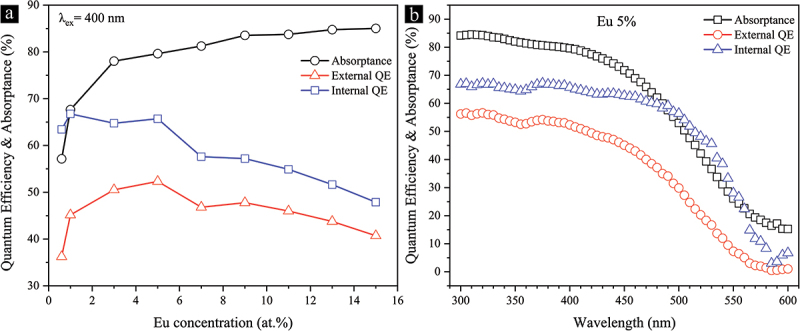
Internal and external quantum efficiencies and absorptance (%) of the single-phase Eu^2+^-doped Sr_3_Si_24_Al_6_N_40_ phosphor powders as a function of (a) Eu^2+^ concentration and (b) the excitation wavelength.

The dependence of internal QE, external QE, and absorptance (%) on the Eu^2+^ concentration and excitation wavelength is investigated and summarized in Figure 13. Absorption increases monotonically with the rise of Eu^2+^, which is consistent with the observed broadening and enhancement in both the high- and low-energy portions of the PLE spectrum (Figure 10b). Internal and external QEs have their highest values of 66% and 52% at 5% Eu^2+^ under 400 nm excitation, respectively. They are comparable to or superior to those of Ca-α-SiAlON:5% Eu^2+^ [98]. Further improvement of the internal QE value to ~ 73–78% of the commercial Ca-α-SiAlON:Eu^2+^ powders can be expected by optimizing the particle size and reducing surface defects [99,100].

**Figure 14.**
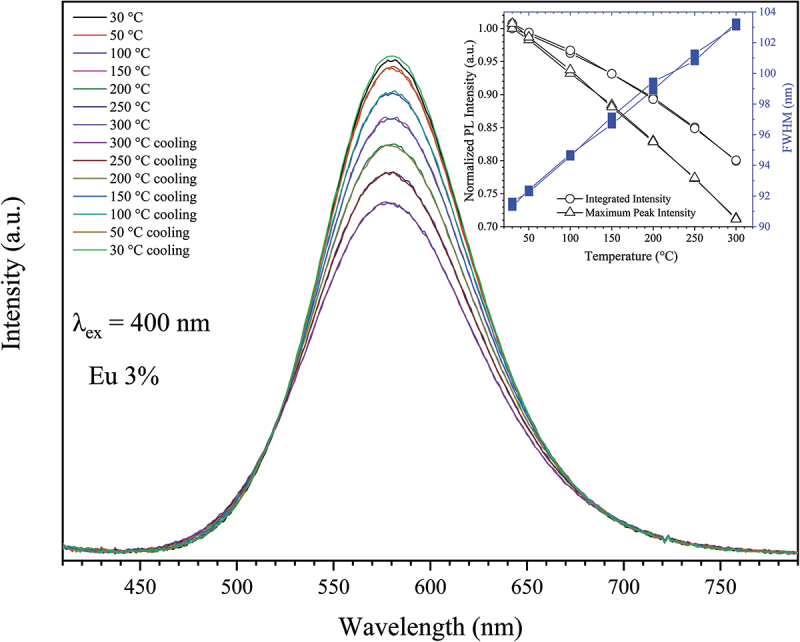
Temperature-dependent PL spectra of the single-phase 3% Eu^2+^-doped Sr_3_Si_24_Al_6_N_40_ phosphor powder in both heating and cooling paths, recorded in ambient conditions. The inset displays the changes of the calculated normalized integrated intensity, peak intensity, and FWHM with the temperature in both heating and cooling paths.

Thermal stability is another crucial factor in determining the usability of a phosphor for high-power pc-wLED applications [101]. Figure 14 shows the normalized PL spectra of the 3% Eu^2+^-doped powders, recorded from 30 to 300°C under 400 nm excitation at ambient conditions. The PL intensity decreases linearly with temperature, reaching 93 and 80% of the RT intensity at 150 and 300°C, respectively. A small reversible blue shift by ~ 3–4 nm in the emission wavelength is observed at 300°C, which can be attributed to the expansion of the coordination environments surrounding the Eu^2+^ ions. The shape and position of the PL band remain unchanged after the heating cycle. Such a promising small degree of thermal quenching is comparable to Ca-α-SiAlON:Eu^2+^ [71] and superior to Sr-α-SiAlON:Eu^2+^ (Sr_0.375_Al_0.77_Si_11.25_N_15.98_O_0.02_: Eu^2+^) [72]. The luminescence quenching can be generally explained by the thermally induced phonon-assisted tunneling of electrons within the 5*d* excited states of the Eu^2+^, followed by nonradiative relaxation to the bottom of the 4*f* ground state [71,72,102]. The thermal ionization of the 5d electron to the host lattice conduction band, as proposed by ten Kate et al., may also be a viable mechanism [103]. To gain a more detailed understanding of the exact mechanism, further research will be required.

**Figure 15.**
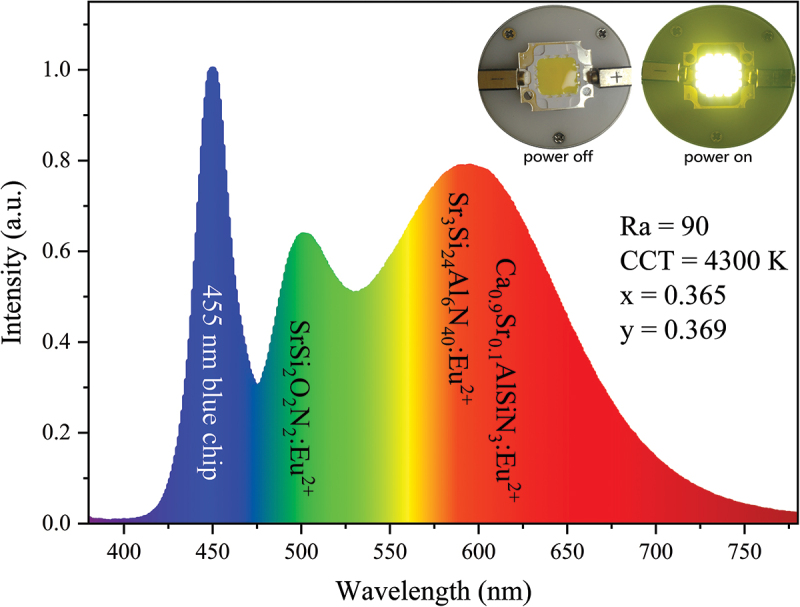
EL Spectrum of a wLED device consisting of a blue LED chip (455 nm) and a mixture of our new yellow-emitting 5% Eu^2+^- doped Sr_3_Si_24_Al_6_N_40_, green-emitting SrSi_2_O_2_N_2_:Eu^2+^, and red-emitting Ca_0.9_Sr_0.1_AlSiN_3_:Eu^2+^ phosphor powders. The appearances in both on and off conditions are shown in the inset.

Finally, we evaluated the potential of this phosphor for solid-state wLED applications. A simple wLED device was fabricated by using a blue LED chip (455 nm) and a mixture of the orange-yellow-emitting Sr_3_Si_24_Al_6_N_40_:5% Eu^2+^, green-emitting SrSi_2_O_2_N_2_:Eu^2+^, and red-emitting Ca_0.9_Sr_0.1_AlSiN_3_:Eu^2+^ powders. As seen in Figure 15, the wLED produces a natural white color with a correlated color temperature (CCT) of 4300 K, a high general CRI (Ra) of 90, and chromaticity coordinates of x = 0.365 and y = 0.369.

## 4. Summary

A single-phase Sr-rich polytypoid α-SiAlON phosphor, Sr_3_Si_24_Al_6_N_40_:Eu^2+^, was synthesized using a remixing-reannealing process at 2050°C under 0.92 MPa N_2_ pressure. The Sr_3_Si_24_Al_6_N_40_ host possesses three distinct Sr sites for Eu^2+^, leading to a broad emission band centered at 590 nm. The Sr_3_Si_24_Al_6_N_40_:Eu^2+^ shows a concentration quenching at 3% Eu^2+^, and its emission intensity only declines by 7% at 150°C, showing a quite high thermal stability. The orange-yellow-emitting phosphor (5% Eu^2+^) exhibits internal and external QEs of 66% and 52% under 400 nm excitation, respectively, which is superior to the reported Ca-α-SiAlON:Eu^2+^. It also demonstrates that the title phosphor would be a promising orange-yellow down-conversion luminescent material for white LEDs. The experimental confirmation of the existence of such ‘Sr-rich’ SiAlON systems, in a single-phase powder form, paves the way for the design and synthesis of novel ‘Sr-rich’ SiAlON-based phosphor powders with unparalleled properties.
